# Lifestyle changes are burdensome with my body broken by pain and obesity: patients’ perspectives after pain rehabilitation

**DOI:** 10.1186/s12891-023-06961-2

**Published:** 2023-10-25

**Authors:** Elin Storm, Nina Bendelin, Kim Bergström Wessman, Maria M Johansson, Mathilda Björk, Huan-Ji Dong

**Affiliations:** 1 Pain and Rehabilitation Centre, and Department of Health, Medicine and Caring Sciences, Linköping University, Linköping, Sweden; 2 Department of Activity and Health in Linköping, and Department of Health, Medicine and Caring Sciences, Linköping University, Linköping, Sweden

**Keywords:** Chronic pain, Obesity, Lifestyle, Interdisciplinary Pain Rehabilitation Program (IPRP), Rehabilitation

## Abstract

**Background:**

Despite the existing evidence regarding the interrelated relationship between pain and obesity, knowledge about patients’ perspectives of this relationship is scarce, especially from patients with chronic pain and obesity after completing Interdisciplinary Pain Rehabilitation Program (IPRP).

**Aims:**

This qualitative study expands the understanding of patients’ perspectives on how chronic pain and obesity influence each other and how the two conditions affect the ability to make lifestyle changes.

**Method:**

A purposive sample of patients with Body Mass Index (BMI) ≥ 30 kg/m^2^ and who had completed an IPRP were recruited for individual semi-structured interviews. The transcribed interviews were analysed using latent content analysis and a pattern of theme and categories was constructed based on the participants’ perspectives.

**Results:**

Sixteen patients (aged 28–63 years, 11 female, BMI 30–43 kg/m^2^) shared their experiences of chronic pain, obesity and lifestyle changes after IPRP. The analysis revealed one overall theme (*lifestyle changes are burdensome with a body broken by both pain and obesity*) and four categories (*pain disturbing days and nights worsens weight control*, *pain-related stress makes lifestyle changes harder*, a *painful and obese body intertwined with negative emotions* and *the overlooked impact of obesity on chronic pain*). Most participants perceived that their pain negatively impacted their obesity, but they were uncertain whether their obesity negatively impacted their pain. Nevertheless, the participants desired and struggled to make lifestyle changes.

**Conclusion:**

After IPRP, patients with chronic pain and obesity perceived difficulties with self-management and struggles with lifestyle changes. They experienced a combined burden of the two conditions. Their perspective on the unilateral relationship between pain and obesity differed from the existing evidence. Future tailored IPRPs should integrate nutritional interventions and address the knowledge gaps as well.

**Supplementary Information:**

The online version contains supplementary material available at 10.1186/s12891-023-06961-2.

## Introduction

There is a complex and not fully understood relationship between pain and obesity [1, 2]. Chronic pain affects everyday life in many aspects, such as sleeping habits, physical activity, daily work, and social life [3, 4]. Obesity, especially severe obesity, is associated with impaired quality of life and increased mortality [5, 6]. A growing body of evidence indicates that the two conditions are interrelated, so it is important to analyse lifestyle factors in patients with chronic pain [7–11].

Some lifestyle factors significantly affect patients suffering from chronic pain and obesity, including physical activity [4, 12, 13], dietary habits [14–17], state of stress [7, 11, 18], sleep routines [7, 17], smoking [19], and alcohol consumption [20]. Available evidence on underlying mechanisms of the connection pain-obesity-lifestyles suggests an important role of lifestyles in occurrence and development of the two conditions. For example, pain-related inflammation, homeostatic balance, and metabolism have been reported to contribute to altered dietary behaviours, which is also closely related to the occurrence of obesity [15]. Obesity affects chronic pain via bio-mechanical load, gut-micorbiome and inflammation and lifestyle factors such as physical (in)activity, stress and sleep disturbance link this relationship [7]. Besides its connection to the two conditions,, lifestyle habits should also be discussed in a broader scope, in light of social and environmental factors as well as personal factors [9, 21]. The current evidence on how lifestyle habits interplay with pain and obesity allows us to believe that lifestyle-related diseases such as chronic pain and obesity should not simply account for a personal choice of lifestyle habits and be blamed on one’s behaviours. Pain and/or obesity therefore could make it difficult to adopt a healthy lifestyle also impact how individuals develop and maintain non-optimal habits. However, knowledge in patients’ perspective on how the two conditions together affect their lifestyle habits is still lacking. An understanding of existing research evidence as well as the perspectives of people with chronic pain and obesity could help develop tailored interventions.

According to the International Association for the Study of Pain (IASP), pain management should consider using Interdisciplinary Pain Rehabilitation Program (IPRP) for patients with complex chronic pain. This clinical practice is based on a bio-psycho-social model and consists of synchronized treatments from different modalities provided by healthcare professionals working in an multidisciplinary team [22, 23]. In Sweden, this patient group is often referred to a specialist pain rehabilitation clinic for pain management due to the complexity of the issue [23, 24]. Comorbidity is one aspect of the complexity and obesity is an extremely common comorbidity [25]. Overall, healthcare professionals often address chronic pain and obesity as separate issues [26], so weight reduction is not a goal in current IPRPs. Numerous studies have shown an interrelated relationship between pain and obesity [12, 13, 27–32], but knowledge is scarce about patients’ experiences living with chronic pain and comorbid obesity. A few studies conducted in primary care clinics and weight management services have investigated patients’ lived experiences and understanding of the two conditions [4, 16, 33]. However, chronic pain was not a major health concern for the included participants. Knowledge is lacking about obese patients’ experiences of participating in pain rehabilitation. A broader understanding is important for clinicians to adapt patient-tailored IPRPs. Thus, this qualitative study focused on the experiences of obese patients who participated in IPRPs in specialist pain rehabilitation clinics. Here, we explored patients’ perspectives on how chronic pain and obesity influenced each other and how the two conditions affected the ability to make lifestyle changes.

## Methods

In this qualitative study, data were collected through semi-structured individual interviews. Data were analysed using latent content analysis with an inductive approach to identify a wide range of experiences, similarities and nuances [34]. The inductive approach was chosen since there was a lack of previous relevant models in this field.

This study was approved by the Swedish Ethical Review Authority (Dnr: 2021/028 − 11).

### Participants

We included 16 patients (i.e., participants) with chronic pain and obesity (Body Mass Index (BMI ≥ 30 kg/m^2^) who completed an IPRP between 2019 and 2021. BMI was calculated based on self-reported body height and weight. The participants were selected via the Swedish Quality Registry for Pain Rehabilitation (SQRP), which stores data from all patients referred to specialist pain clinics in Sweden. Patients from two clinics in south-east Sweden were invited to participate in the study.

IPRP is conducted in groups of six to eight patients, lasts between 6 and 8 weeks, and includes patient education, supervised physical exercise, training in simulated environments, and cognitive behavioural therapy [35]. The interdisciplinary rehabilitation team includes a psychologist, physiotherapist, occupational therapist, and a physician specialized in rehabilitation. The interdisciplinary team of professionals strives for a close collaborative approach with the patient and considers the patient’s specific goals. Each patient sets individual rehabilitation goals and makes an individual schedule in collaboration with the rehabilitation team [35].

SQRP collects information on patients’ socio-demographic background (i.e., gender, age, country of birth, education, occupational status, etc.), pain characteristics, and pain-related psychosocial aspects before, directly after, and one year after the IPRP. Pain intensity during the last seven days was measured using the Numerical Rating Scale (NRS-7 days), which is a 11-point scale ranging from ‘no pain’ to ‘worst imaginable pain’ [36]. Pain spreading was measured using the Pain Region Index (PRI) where patients reported which of the 36 predefined anatomical areas (18 on the front and 18 on the back of the body) they feel pain [35, 37]. Anxiety and depressive symptoms were measured using the Hospital Anxiety and Depression Scale (HADS) [38]. The scoring ranges from 0 to 21 and a higher score indicates higher possibility of anxiety or depression. Sleep disturbance was measured using the Insomnia Severity Index (ISI) [39, 40]. ISI is composed of seven items evaluating characteristics of insomnia in the last two weeks, with a total score range from 0 to 28, where high scores indicate more severe insomnia. A summary of the participants’ socio-demographic background, pain characteristics, and pain-related psychosocial aspects is presented in Table 1.

**Table 1:** Characteristics of included participants (N = 16) before participating in IPRP

Variables	Values	Min-Max
Gender female/male, n	11/5	
Age, years, Mean ± SD	43.8 ± 10.2	28–63
Body Mass Index, Mean ± SD	35.7 ± 4.4	30–43
Born in Sweden, n	16	
Education Levels, n		
Elementary school	1	
Upper secondary school	7	
University	7	
Other	1	
Current working ≥ 25%, n	11	
Pain intensity (scale 0–10), Mean ± SD	5.5 ± 1.7	2.5–8
Pain duration, years, Median (Q1-Q3)	8 (4.4–20.8)	
Pain distribution (Range 0–36), Median (Q1-Q3)	19 (14-26.5)	2–34
Pain diagnosis		
Fibromyalgia/widespread pain	5	
Lower back pain	4	
Hypermobility syndromes	4	
Joint pain	2	
Myalgia (not specified)	1	
HADS-anxiety, Mean ± SD	9.9 ± 5.0	2–20
HADS-depression, Mean ± SD	11.0 ± 4.0	4–18
Insomnia Severity Index, Mean ± SD	15.6 ± 8.7	1–27

Notes: SD: standard deviation; Q1: 1st quartile; Q3: 3rd quartile; HADS: Hospital Anxiety and Depression Scale.

### Data collection

To ensure data saturation, we recruited participants consecutively in parallel with analysis of interview transcripts. The researchers discussed and reached a consensus across views that no new information in relation to the study aim was retrieved in the last interview. Eligible participants were identified via the SQRP based on the study criteria of BMI levels and IPRP completion. Invitation letters, which included a project description and contact information, were sent to five potential participants at a time. These potential participants were recruited from the two pain clinics. In a telephone conversation with potential participants, a research assistant provided more details about the interview process and assessed their interest in participation. If they expressed an interest to participate, an interview was scheduled. Participation was voluntary and the participants were not offered any economic compensation. An informed consent was signed before the interview started. After each set of interviews was completed, the research group discussed the transcripts, and five additional invitation letters were sent out for further recruitment. In total, 26 eligible participants were invited. Three could not be reached by telephone, six declined participation due to language difficulties (n = 2), lack of time (n = 1), health issue (n = 1), or through e-mail without stating a reason (n = 2). One person was omitted for failing to attend the scheduled interview.

Semi-structured individual interviews were conducted over two periods: September–November 2021 (by ES) and February–March 2022 (by KBW). The locations for the interviews were chosen by the participants. Due to the Covid-19 pandemic, most interviews (n = 12) were performed remotely using secure video calls (software provided by the healthcare service to protect patients’ privacy and confidentiality). The remaining (n = 4) were interviewed on site at their pain clinic. The interviews lasted between 32 and 65 min (mean 50 min) and were audio-recorded and transcribed verbatim. The interviewers (ES and KBW) had not been involved in the participants’ rehabilitation.

A semi-structured interview guide was developed by HJD and MMJ focusing on three areas: *Pain and lifestyle habits*, *Lifestyle habits after IPRP*, and *Relationship between pain and body weight*. We addressed the lifestyle habits related to diet, exercise, sleep, tobacco, alcohol, and stress. After two pilot interviews by ES, a few probing questions were added to encourage participants to freely express and elaborate their experiences – e.g., Would you please to say something more about this experience? Why do you think it is like this? In your opinion, how has pain affected this?

### Data analysis

Interviews were transcribed verbatim by the interviewers (ES and KBW) or an experienced research assistant. First, the research group read the transcribed texts several times to gain a general impression. Second, meaning units relevant to the aims of the study were identified and coded. Third, codes were formulated and sorted into subcategories by comparing and appraising the codes to determine which codes seemed to belong together. The subcategories were then abstracted to categories. Finally, a theme emerged that represented a response pattern important to the study aim and relevant for all participants [34]. Table 2 provides some examples of the analysis process, from meanings of unit to the theme. More examples in each subcategory are given in Additional Table 1.

**Table 2:** Examples from the analysis

Meaning unit	Condensed meaning unit	Code	Subcategory	Category
*You don’t have the same eating habits as you had before when you worked, I would say.*	Changed eating habits at work	Sick leave affects eating habits	Altered daily routines disrupt lifestyles and weight control	Pain disturbing days and nights worsens weight control
*When a severe pain period kicks in, I become stressed and it’s like I black out.*	Severe pain causes stress and blackout	Pain causes stress	Unpredictable pain disrupts planned activities	Pain-related stress makes lifestyle changes harder

The analysis process was mainly conducted by two of the authors (ES and KBW), with continuous discussions in the research group. The first author (ES) performed the categorization with consensus discussions with the research group (NB, KBW, MMJ, MB, and H-J D), consisting of researchers with different clinical professions (medical student, physician, occupational therapists, and psychologist) and with various research experience. The research group repeatedly discussed the evolving findings in the research group to reach a comprehensive understanding [41]. Different interpretations of the data were frequently discussed that led to insightful illustrations and a final mutual understanding of the results. The data were deemed saturated after multiple checks (ES, NB, MB, and HJD) of the richness and thickness of the data and the similarities and variations of the participants’ experiences [42].

## Results

The analysis resulted in one theme, four categories, and nine sub-categories (Table 3).

**Table 3:** Overview of the subcategories and categories

Subcategories	Categories	Theme
Altered daily routines disrupt lifestyles and weight control	Pain disturbing days and nights worsens weight control	Lifestyle changes are burdensome with a body broken by both pain and obesity
Impaired sleep comes with a sedentary lifestyle		
Pain takes away energy and time from me	Pain-related stress makes lifestyle changes harder	
Unhealthy food more accessible at home increasing eating		
Unpredictable pain disrupts planned activities		
Sedentariness due to a broken body	A painful and obese body intertwined with negative emotions	
Emotional regulation by eating and smoking		
Hesitant on obesity affecting pain	The overlooked impact of obesity on chronic pain	
Unbelievable that obesity causes pain		

### Theme: Lifestyle changes are burdensome with a body broken by both pain and obesity

This theme refers to the participants’ perspectives on a struggle for lifestyle changes as they were living with chronic pain and comorbid obesity. Pain appeared to have a general negative impact on the participants’ lifestyle habits, sometimes to such extent that it altered lifestyle habits that might have caused weight gain. Their altered daily routines appeared to have negatively influenced their eating habits and physical activity, which challenged their ability to control their weight. They seemed not to have recognized or expressed uncertainty towards an effect of obesity on pain, as they perceived the relationship between pain and body weight as primarily unilateral, where pain could potentially lead to obesity. They desired and struggled to achieve weight control or weight loss. However, in their multiple attempts to make lifestyle changes, pain appeared to always be present and disrupt the progress.

The theme shed light on the complex interaction between pain and obesity, where lifestyle habits appear to play a significant role. The following categories and sub-categories illustrate the participants’ experiences of how pain and obesity affected their lifestyle habits.

### Pain disturbing days and nights worsens weight control

This category describes the participants’ experiences of how pain interfered with their everyday life, during the day as well as at night, leading to unfavourable conditions for weight control.

#### Altered daily routines disrupt lifestyles and weight control

Participants seemed to be aware of the importance of balancing calorie intake and expenditure to achieve weight control. However, they described the difficulty of keeping this balance as pain was perceived to have made it difficult to maintain daily routines and lifestyle habits. For example, pain could hinder the ability to engage in physical exercises, which used to be one of the hobbies. Sick leave caused by pain appeared to have a major impact on daily routines and negatively altered lifestyle habits.

Unhealthy eating and poor sleep were perceived as consequences as well as decreased physical activity due to more time staying at home. Participants explained their own approaches to improve diet patterns based on their knowledge. They tried diets such as reducing sugar intake to alleviate pain caused by inflammation. It also seemed obvious that more sedentary days caused by pain led to weight gain and/or difficulties losing weight:

*Since I don’t get moving if not necessary, I become sedentary or lay on the couch. As I don’t move, I don’t burn anything of what I have eaten. Therefore, I weigh more. (Participant 1)*

*Impaired sleep comes with a sedentary lifestyle: Pain* affected participants’ sleep, so they put effort and money into improving sleep quality. They had bought a new bed mattress. They also took sleep medications and/or pain medications.

Participants perceived that their impaired sleep was related to daily sedentariness, which was caused by chronic pain. They needed a significant amount of time for frequent rests (i.e., they needed breaks without any physical or mental effort) and even naps. In contrast, one participant experienced that he could improve his sleep by being physically active during the day.

Poor sleep appeared to affect the participants’ functional ability during the day. Being awake at night also affected their eating habits, which led to an increased calorie intake during the night:

*If I don’t sleep, I will likely eat something. Eh. . partly because my body’s signals just tell me to go to the refrigerator. (Participant 12)*

### Pain-related stress makes lifestyle changes harder

Participants described how pain led to increased everyday stress, which affected their lifestyle and made lifestyle changes more demanding. Lifestyle habits seemed to be affected in a discouraging manner even on the days they did not experience severe pain or had manageable pain. They experienced this to be a result of their overall increased stress level.

#### Pain takes away energy and time from me

Participants experienced that pain drained their overall energy. Simultaneously, lifestyle changes were perceived as time consuming and requiring significant effort. This unexpected barrier disturbed their efforts to prepare and cook healthy meals. As a result, they avoided everyday activities perceived as too burdensome.

*My husband cooks. I can’t take it or handle it. That is why I give up and we eat worse food instead. (Participant 3)*

The reduced energy decreased their everyday activity level. Activities were perceived as more time consuming than before. The lack of time appeared to be a major factor for stress as was the extra effort needed to do things right. They had made a habit of pacing their time and energy on a few activities a day regardless of pain intensity:

*If I try to go for a walk in the forest or something, I must keep in mind that I have. . I usually call it ‘my pain energy’. . my energy volume is like five pieces of cake, which I can distribute every day. . ., for example, grocery shopping consumes one piece. (Participant 5)*

#### Unhealthy food more accessible at home increasing eating

Unhealthy food and sweets were more easily accessible during the day as well as when awake at night and did not require as much time to cook.

*After I have been on foot the whole day and have been to work, I am immensely tired. Then it’s like the whole body screams it’s weary and wants energy. . fast, fast carbohydrates, candy, or chocolate. (Participant 14)*

Participants mentioned a preference for food with high calories/low nutrients. Moreover, when they were sedentary, they were more likely to consume this type of food.

#### Unpredictable pain disrupts planned activities

Pain was perceived as unpredictable, and this could help explain why participants felt stressed. Participants experienced difficulties planning future activities because pain could suddenly worsen. It was discouraging and made them doubt their ability to do everyday activities including cooking. Hence, they sometimes chose meals with less preparation time/cooking time.

*It’s not practical to stand up and make a slow cook or a stew or something that takes longer time. Instead, I limit myself to do the things that I will be able to finish before the pain kicks in. (Participant 1)*

### A painful and obese body intertwined with negative emotions

This category describes the experience that pain is constantly present in a body with comorbid obesity and pain, which affects physical and mental health. Participants expressed frustrations with attempts to increase their physical activity and to cope with a painful and obese body.

#### Sedentariness due to a broken body

The term ‘broken’ referred to the perception of a dysfunctional body compared to previous physical ability or compared to the participants’ expectations of physical capacity. Participants considered their body damaged or in risk of damage. Physical activity seemed to have been a previously enjoyable activity for some participants. Pain had taken away the joy of and motivation to be physically active. Pain could be an excuse to avoid physical activity although the pain sometimes did not stop them from being somewhat active.

*Can’t we go for a walk? My husband asked. Ah, no, I don’t want to, so I sit down. Knits or crotchet is what I like very much. So, I blame it on my pain, even though I might not be in huge pain right then [. .]. (Participant 7)*

Pain led to reduced working capacity and a need for less physically demanding work tasks. Thus, the consequence was a more sedentary lifestyle, not only at home but also at work.

Participants strived for a more active lifestyle to relieve pain, but at the same time pain could hinder them from engaging in physical activities. Whether to use pain medications was a dilemma for these participants. They perceived that pain medications were crucial to perform physical activity, but they also expressed concerns about side effects of their medications, primarily relating to weight gain.

#### Emotional regulation by eating and smoking

Participants looked for ways to distract themselves from the pain. This distraction led to different choices. A common reflection was the strategy of committing to ‘good discipline’, especially when unhealthy foods were easily accessible as a ‘quick fix’ to distressing emotions. Excessive calorie intake was partly caused by emotional eating, induced by pain-related mood or stress. Food, including sweets, seemed to distract participants from pain and delight them.

*When you are in pain you can’t do so much. . then I shall have something nice or cosily instead. Then it’s easy to use food or something else, hmm. . candy. (Participant 2)*

In addition to feeling bad or painful, stress was also described as a feeling that could lead to unhealthy eating behaviours:

*I am doing things slowly due to my low energy level, and it makes me feel stressed. The stress drives me to eat unhealthy food. (Participant 5)*

It was mentioned that pain increased their smoking as it distracted them from pain-induced emotions and pain-focused thoughts or provided comfort:

*When you can’t use your body, you get frustrated and restless [. .]. When I am in such a situation and want to break the pattern, it happens that I go out and smoke a cigarette. (Participant 11)*

However, alcohol consumption was almost non-existent or very low among most participants. Only one participant tried using alcohol to fall asleep.

### The overlooked impact of obesity on chronic pain

This category described that participants did not always recognize an effect of obesity on pain, as they perceived the relationship between pain and body weight as primarily unilateral, where pain could potentially lead to obesity. Two sub-categories emerged: *Hesitant on obesity affecting pain and Unbelievable that obesity causes pain.*

#### Hesitant on obesity affecting pain

Although it was acknowledged that excess body weight led to pain as the result of mechanical stress on joints and the spine, participants were generally hesitant towards whether high body weight could affect pain. The hesitation was existing despite that the impact of excess weight on pain seemed to have been recognized in daily activities.

*Of course, it would have been great for the pain not having to carry this weight. [. .] I work in childcare, and I rarely run nowadays. But sometimes I must run at work. . and then, my pain is always extremely bad the next day. . the heavy impact, or what to say. So, it [weight] does affect my pain. (Participant 14)*

#### Unbelievable that obesity causes pain

It was also unimaginable that high bodyweight could affect pain.

*I have friends who are much slimmer than I, err. . very lean,. . who have just as much pain as I, even though they are lean. So, I don’t think, mmm, I don’t know. I am trying to figure out some sort of connection. It’s possible that there is one, but I don’t know yet. (Participant 4)*

Participants with pain debut after an accident or a surgical intervention did not relate obesity to pain. Surprisingly, participants who had experienced pain relief after successful weight loss did not believe that obesity could cause pain as they still had pain in other body parts. Yet, some believed that reduced weight could have a positive effect on mobility:

*Overall, it’s easier to move [laughs]. It’s, like, as you say, it does not hold you back from tying your shoelaces anymore. This effect alone is a positive result. (Participant 12)*

## Discussion

For this qualitative study, we recruited patients who had chronic pain with comorbid obesity and who had participated in IPRPs to obtain knowledge of their health conditions and coping strategies for living with chronic pain. They perceived pain disturbed their daily routines and lifestyles, which challenged their ability to control their weight. They desired and attempted to make lifestyle changes to lose weight. They shared their perspectives on how chronic pain and comorbid obesity signified a struggle to cope with negative emotions. They seemed have overlooked the impact of obesity on chronic pain as they only perceived the unilateral direction, where pain could potentially lead to obesity.

The participants’ perspective of unilateral relationship between pain and obesity differs from the existing evidence [1, 7, 43]. We reflect that it is still not generally acknowledged that chronic pain can be considered as a lifestyle related disease [11]. Knowledge about increased biomechanical loading negatively affecting the musculoskeletal system is to some extent well-known, but knowledge such as low-grade (neuro)inflammation or mediated effects on other pain related lifestyle factors (e.g. sleep, stress, physical activity) warrants to be introduced during the clinical consultations. From a pain rehabilitation perspective, it indicates that patients who completed IPRP did not acquire enough knowledge about the complex interrelated relationship between pain and obesity. Although they had insights on how pain reduction could motivate one to lose weight, they do not acknowledge this relationship as a motivator. This finding contradicts the perspectives from obese patients in another study within a weight management service; these patients were very aware of pain as a primary motivator for weight loss [4]. The finding also suggests that our evidence-based IPRPs need to include specific interventions that make it possible for obese patients to reduce weight and pain simultaneously.

Our participants perceived that pain affected their weight reduction because pain and its negative consequences became barriers to optimizing a healthy lifestyle. As expected, some of these experiences were also expressed in a previous study of patients with obesity [16] or supported by other related studies. For example, their experiences of the relationship between chronic pain and poor sleep quality are in line with the current evidence [7, 44]. They seemed aware that poor sleep might increase the risk of gaining fat [45] as they increased food intake due to sleep disturbance [17]. Our participants shared with us their lived experiences of a dynamic interaction between pain and stress. Given these data, we speculate that their experiences of pain-related stress might be related to more energy expenditure and increased intake of unhealthy food, a conclusion that is supported in the evidence [46].

Pain and obesity appeared to have negative impacts on physical activity levels in terms of physical exercise and everyday physical activity [13]. Our participants’ description of a sedentary life reflects what has been stated in earlier studies – i.e., pain reinforces negative thoughts about being active and creates a vicious cycle of pain-inactivity-weight gain-more pain. This also sheds light on how chronic pain can be a barrier to weight loss by obstructing increased physical activity [4, 12]. Altered daily routines (sleeping, working, and leisure activities) contributed to a more sedentary lifestyle and access to unhealthy food. Existing knowledge clearly states that a sedentary lifestyle is associated with weight gain, which involves fat accumulation that leads to structural and functional changes in tissues, organs, and body systems [47]. Notwithstanding pain-related routine changes, the overall modern western lifestyle (significant sedentariness and easy access to energy condensed food) may also play a significant role. Pontzer and colleagues found abnormally low metabolic rates among people living a western lifestyle by comparing the daily total energy expenditure of Hadza hunter-gatherers and western counterparts [48].

Eating behaviour and dietary intake have shown bilateral relationships with chronic pain [14, 15]. For example, pain could be a negative stressor for emotional eating [16, 17], which was also described by our participants. This behaviour was described as an act of self-comfort and a pleasure in their lives. Healthy dietary intake may influence chronic pain experiences [49]. It has been found that through antioxidant and anti-inflammatory qualities, different diets seem to influence chronic pain [14]. Such knowledge and patient-tailored interventions on especially emotional eating need to be warranted in future IPRPs.

Surprisingly, use of tobacco and alcohol appeared to be modest among these individuals. None of the participants used this prominent coping strategy for chronic pain. After participating in IPRP, patients are expected to be informed and to practice relevant coping strategies to handle pain in their everyday life. Tobacco consumption has been found as a common coping mechanism for pain-induced anxiety [10]. We did not find any information about efforts on smoking cessation after IPRP. Regarding high alcohol consumption, no concern was stated by any participant. One reasonable explanation may be the exclusion criteria stating that persons with a problematic alcohol consumption are not eligible for IPRP [37].

### Strengths and limitations

To our knowledge, this is the first qualitative study focusing on obese patients who completed IPRP at a pain rehabilitation centre. A purposive sampling for this study allowed for inclusion of participants with different perspectives, which strengthens the credibility of the study [50]. We chose individuals from two rehabilitation clinics. The participants were heterogenous with respect to gender, education level, working situation, and pain features. Their perspectives elicit new knowledge that could help healthcare professionals improve current IPRPs to better suit patients with comorbid obesity.

Another strength is our multi-professional research group with a background in medical and health research field, and two (NB and H-JD) have significant clinical experience from this patient group. The interviewing authors (ES and KBW) have medical knowledge but no prior clinical experience, which decreased the risk of their pre-understandings affecting the interview situation [41]. All categories and subcategories were discussed back and forth with experienced qualitative researchers (MB and MMJ) to achieve consensus. This as well as the description of participants and settings strengthens the dependability of the study. To ensure confirmability we described the analyses procedure and gave examples of the process in Table 2.

This study has some limitations. One limitation is its transferability. Participants were referred to a specialist pain rehabilitation centre due to complex pain conditions with moderate to severe psychological and/or somatic comorbidities and in need of specialist care [25]. This specific context needs to be considered. Therefore, our findings may not be transferable to other clinical settings where patients have less complex or mild clinical presentations (i.e., obesity might not be a common comorbidity). Another limitation is that we are unaware of the perspectives of the potential participants who declined our interviews. One important barrier could be weight stigma, which is extremely common in this patient group [51]. Therefore, a possible selection bias might have occurred in individuals who were open about their bodyweight issues and their need to consult healthcare professionals.

### Relevance to clinical practice

This study contributes to a broader understanding of existing challenges in lifestyle changes among patients with chronic pain and obesity. Nutritional interventions need to be integrated in IPRPs to benefit patients with comorbid obesity. Healthcare professionals need to be prepared to discuss and provide a clear description of the relationship between pain and obesity. The relationship between pain and obesity could be established by developing patient education [26] and providing multidisciplinary interventions based on a coherent rational for the relationship between lifestyle, pain, and obesity based on the existing evidence [49]. Current IPRPs may help obese patients tackle certain weight management challenges such as a sedentary lifestyle, emotional distress, and catastrophizing [31]. However, an effective way to relieve pain is to treat both conditions simultaneously. The current IPRPs in Sweden rarely include a dietitian, who typically has a crucial role for providing nutritional interventions in weight management. In future patient-tailored IPRPs, we recommend that nutritional interventions should include screening, nutrition education, dietary recommendations, and evaluations and be integrated for patients with comorbid obesity to enable successful lifestyle changes and pain management [49]. Moreover, as our participants experienced emotional eating, patient-tailored IPRPs should also consider psychological, especially behavioural treatments, targeting emotional eating [43, 49]. Patients with severe eating disorders ought to be referred to psychiatric specialist care to alleviate mental health.

## Conclusions

Patients’ perspectives on the unilateral relationship between pain and obesity differed from the existing evidence. After completing IPRP focusing on pain self-management, patients with chronic pain and comorbid obesity signified a struggle for lifestyle changes due to the combined burden of the two conditions. These findings should help clinicians adapt future patient-tailored IPRPs to address the knowledge gap of the relationship between pain and obesity. Furthermore, there is a need to integrate nutritional interventions to target the eating behaviours.

### Electronic supplementary material

Below is the link to the electronic supplementary material.

Additional Table 1

## Data Availability

The datasets used and/or analysed during the current study available from the corresponding author on reasonable request.
