# Supplementary material for: Lifestyle changes are burdensome with my body broken by pain and obesity: patients’ perspectives after pain rehabilitation
Source: BMC Musculoskelet Disord. 2023 Oct 25;24:840. doi: 10.1186/s12891-023-06961-2 (PMC10599046; doi:10.1186/s12891-023-06961-2)
Supplement: Supplementary file 1 — Additional Table 1 [file 12891_2023_6961_MOESM1_ESM.docx]

**Table S1** Some more examples from the analysis.

| Meaning unit | Condensed meaning unit | Code | Subcategory | Category |
| --- | --- | --- | --- | --- |
| *I feel like …. I sleep better when I have done something active.* | Better sleep when being able to be more active | Sedentariness impair sleep | Impaired sleep comes with a sedentary lifestyle | Pain disturbing days and nights worsens weight control |
| *I was staying home during sick leave, and it was spring and sunny outside, so we sat outside and ate ice cream and such. Then I realized I had gained weight much fast.* | Sick leave caused increased eating and weight gain | Home stay causes weight gain | unhealthy food more accessible at home increasing eating | Pain-related stress makes lifestyle changes harder |
| *The pain takes up so much time that I… I need to spend so much energy on … surviving the pain.* | Energy is spent on surviving the pain | Pain costs energy | Pain takes away energy and time from me |  |
| *When I have pain I do not really want to move, it feels better to sit still.* | Don’t want to move because of pain | Pain restricts movement | Sedentariness due to a broken body | A painful and obese body intertwined with negative emotions |
| *There are moments when you eat more because you feel depressed.* | Eat more due to emotions | Emotional eating | Emotional regulation by eating and smoking |  |
| *I don’t know if weight loss has an impact on my health because it is hard to see connections to my pain.* | Unsure if weight affects health. Unclear connection with pain | Unclear connection between weight and pain | Hesitant on obesity affecting pain | The overlooked impact of obesity on chronic pain |
| *No, I cannot say that it is the pain that has an impact on the pain.* | Cannot see the effect of weight on pain | Weight doesn’t affect pain | Unbelievable that obesity causes pain |  |
